# Carbolic Acid as an Obtundent

**Published:** 1888-03

**Authors:** 


### CARBOLIC ACID AS AN OBTUNDENT.

How many dentists are in the habit of employing carbolic acid for the purpose of overcoming the sensitiveness of a dental pulp, or that of the nerve canal? And yet we believe it the best agent for that purpose which is known to the dental pharmacopœia. A pulp may be painlessly removed by its use if patience be exercised. The extreme sensitiveness that sometimes exists at the ulterior point of a canal may be entirely removed if a little time and care are used in working the agent to the sensitive point. The rubber-dam must of course be first applied, and then a delicate broach, which is frequently dipped in the carbolic acid, should be carefully worked into the cavity, and gradually advanced as the obtunding process goes on. If the cavity or canal be large enough, a film or two of cotton may be wound around the broach. The best instruments for this purpose that we have ever used are the Donaldson broaches, and canal cleaners. If one of the latter can be employed, it will, simultaneously with carrying the carbolic acid to place, remove all of the obtunded pulp.

